# External Load During Warm-Up and Early-Match Running Performance of Starting and Substitute Football Players: A Within-Player Comparison

**DOI:** 10.3390/jfmk11040432

**Published:** 2026-10-19

**Authors:** Gabriele Bagattini, Luis Suarez-Arrones, Manuele Ferrini, Jose Asian-Clemente

**Affiliations:** 1 Department of Sport Sciences, Universidad Pablo de Olavide, 41013 Sevilla, Spain; g.bagattini@me.com (G.B.); manuele.ferrini@gmail.com (M.F.); jasicle@upo.es (J.A.-C.); 2 Performance and Health Department, FC Lugano, 6900 Lugano, Switzerland; 3 FSI Lab, Football Science Institute, 18016 Granada, Spain

**Keywords:** football, soccer, warm-up, substitute players, starter players, external load, GPS monitoring, high-intensity activity, running performance, variability

## Abstract

**Objectives**: This study aimed to: (1) compare the absolute and relative external load during the warm-up of starting and substitute professional football players; (2) describe the subsequent running performance during the first 15 min of match play; and (3) quantify the within-player variability of the warm-up (WU) external load between roles. **Methods**: Sixteen professional male players (Swiss Second Division) were monitored using 10 Hz GPS units across 13 official matches (106 observations). Starters completed a standardized ~25 min on-field warm-up, while substitutes performed a cyclical sideline warm-up. Running performance was analysed during the first 15 min of play and across three consecutive 5 min intervals (0–5, 5–10, and 10–15 min) in the same players under both match-entry conditions: starters and substitutes. **Results**: Starters’ warm-up showed significantly longer duration (*p* < 0.001; *d* = 3.5), greater total distance covered in both absolute and relative terms (*p* < 0.001, *d* = 6.6; *p* < 0.001, *d* = 5.1, respectively), and higher maximal speed (*p* < 0.001; *d* = 1.4) compared to substitutes. However, substitutes covered more distance between 19.8 and 25.2 km·h^−1^ in either absolute and relative terms (*p* < 0.05, *d* = 1.3; *p* < 0.001, *d* = 3.6; respectively). No significant differences were found in external load during the overall 0–15 min match period between roles. Nevertheless, block-specific analysis revealed that substitutes performed significantly a greater number of decelerations (*p* < 0.05; *d* = 0.5), distance covered between 19.8 and 25.2 km·h^−1^ (*p* < 0.05; *d* = 0.79) and sprinting during the initial 0–5 min period (*p* < 0.05; *d* = 1.24). **Conclusions**: Substitutes’ warm-up exhibited substantially higher variability across almost all external load metrics compared with the warm-up completed by the starters, except for slightly higher or identical variability in the number of accelerations and decelerations.

## 1. Introduction

Football is a sport physically characterized by the frequent occurrence of intermittent and repeated high-intensity actions [1]. During a match, players are required to perform approximately 1.200 non-cyclical and unpredictable activity changes, typically occurring every 3 to 5 s [2]. These actions prominently include repeated efforts such as sprints, accelerations, decelerations, kicking, dribbling, jumping, and tackling [3]. The importance of these high-intensity actions is underscored by evidence indicating that the vast majority of goals (83%) are preceded by at least one explosive movement, such as a straight-line sprint, changes in direction at speed, jumps, or a combination of these actions [4]. Beyond their decisive influence on match outcomes, these high-intensity actions constitute a major component of the physical demands imposed on players. In support of this, a longitudinal study of the English Premier League over seven seasons (2006–2013) reported increases of approximately 30% in high-intensity running distance and 50% in the number of high-intensity efforts performed during matches [5]. These findings illustrate the progressive increase in the physical demands of modern football and emphasize the ability to perform high-intensity actions as a key performance indicator and an essential component of player development [2].

Beyond the capacity to perform isolated high-intensity actions, the ability to sustain and repeatedly execute these efforts throughout a match is equally important [6]. Several studies have reported lower levels of high-intensity running during the early stages of the second half compared with the corresponding period of the first half [7]. Furthermore, professional soccer players typically exhibit progressive declines in both total distance covered and high-intensity activity as the match progresses, particularly during the final stages of competition [2]. These performance decrements are likely multifactorial, reflecting the combined influence of tactical adjustments, accumulated fatigue, the duration of the halftime interval, environmental conditions, and inadequate preparation for the physical demands encountered after the break [8,9]. To mitigate these declines, coaches may introduce substitute players, a strategy that has become increasingly relevant since the Fédération International de Football Association (FIFA^®^) increased the number of permitted substitutions from three to five per match in 2021. This regulatory change has created a distinct performance context focused on substitute players and further emphasizes the need to better understand the factors influencing their performance [10]. Previous research has shown that substitute players often achieve greater physical outputs than those who remain on the pitch for the entire match [11]. Specifically, substitutes cover greater total distance and high-speed distance during the first five minutes following their introduction compared with subsequent five-minute periods [12,13]. Moreover, they tend to perform greater relative high-speed running distance per minute than both starting players and full match players, with this relative impact increasing when substitutions occur later in the game [13]. However, substitutes entering during the final 15 min of matchplay exhibit the lowest peak high-speed running and acceleration demands compared with substitutes introduced earlier in the match [14].

While previous research has largely focused on describing the external load achieved by substitute players relative to starters and full-match players [13,14,15], considerably less attention has been paid to the preparatory processes that may influence these performance outcomes. Among these factors, the warm-up process appears particularly relevant, given its well-established role in optimizing physiological readiness and subsequent running performance. Whereas starting players typically complete a structured warm-up of approximately 30 min, characterized by a progressive increase in intensity and a well-defined pre-kick-off routine, substitutes instead follow more variable, match-dependent preparation routines and often experience prolonged and unpredictable periods of inactivity before entering play [12]. This greater variability and lower degree of standardization may result in substitutes beginning match play in a physiologically underprepared state, with key variables such as core temperature, heart rate, and muscle temperature failing to reach optimal levels [16]. Furthermore, the interval between warm-up completion and competitive activity may also affect subsequent performance. While substitutes generally have only a short period between their final preparatory activity and match entry, starting players are often exposed to extended inactive periods before kick-off. Previous evidence suggests that following 20 min of rest, athletes may experience an approximately 15% reduction in jumping performance [16], whereas 40 min of inactivity may result in declines of around 20% for jumping performance and 6% for sprint performance [17]. Consequently, differences in the timing and structure of pre-competition routines may influence players’ physiological readiness and their ability to perform high-intensity actions immediately after entering the field. In addition to differences in preparation timing, the activities performed before a match entry also differ substantially between player roles. Substitutes’ warm-ups are characterized by very low high-speed running demands, with players covering less than 2 m·min^−1^ at speeds between 19.8 and 25.2 km·h^−1^ and performing no sprinting (>25.2 km·h^−1^) [12]. This limited high-intensity stimulus may further compromise physiological readiness at the time of match entry. Although warm-up strategies incorporating an appropriate combination of duration, intensity, and moderate-to-high intensity activities have consistently been shown to improve repeated-sprint performance during match play [18], the practical constraints of competition often limit their implementation, especially for substitute players. Consequently, these preparatory constraints may influence the running performance achieved during the initial stages of match participation [19].

Despite the practical relevance of this topic, there is still limited evidence regarding warm-up practices in both player roles and their influence on players’ external load during competition. Therefore, it is necessary to better understand the differences in warm-up behaviors between substitute and starting players. In addition, it is also important to examine performance during the initial minutes of match play depending on whether a player acts as a starter or a substitute. Given that the primary purpose of a substitution is to contribute to match performance, preparatory routines should ideally be evaluated based on their impact on subsequent on-field performance. However, previous research has largely relied on indirect measures of readiness rather than actual match performance outcomes. GPS-derived external load provides an objective assessment of the physical demands experienced by starters and substitutes during warm-up and subsequent match participation. Therefore, the present study should be considered an initial descriptive investigation, as it does not assess physiological readiness or the technical, tactical, and match-decisive contributions of players. Accordingly, the aims of this study were: (1) compare the absolute and relative external load during the warm-up between starting and substitute players; (2) describe the external load accumulated during the first 15 min of match play in both groups following the warm-up; and (3) quantify and compare the between-group variability in warm-up external load using the coefficient of variation (CV).

Based on the more structured nature and longer duration of the starting players’ warm-up, we hypothesized that starters would accumulate a greater absolute external load during the warm-up than substitutes. Given the less standardized nature of substitutes’ warm-up routines, we further hypothesized that warm-up external-load variables would exhibit greater variability among substitutes than starters. We hypothesized that overall external load during the first 15 min of match participation would be comparable between starters and substitutes, but that differences would emerge across the three consecutive 5 min intervals.

## 2. Materials and Methods

### 2.1. Participants

Throughout the 2024–2025 season, time–motion data were collected across 13 official league matches with a professional team from the Swiss Second Division (Challenge League). The study sample consisted of 16 players (age = 26 ± 5 yr; height = 180 ± 6 cm, body mass = 74 ± 6.6 kg). All players participated in matches both as starters and as substitutes, contributing data from 7 matches as starters and 11 matches as substitutes. Data were collected through daily monitoring as part of the team’s routine training and match analysis procedures throughout the competitive season. The data were subsequently anonymized and analyzed descriptively. Although, in accordance with previously published recommendations for this type of study, ethical committee approval was considered unnecessary [20]. Nevertheless, the investigation was carried out in line with the ethical standards of the Declaration of Helsinki (2013 revision) [21]. In addition, informed consent was obtained from all participants prior to their involvement. In compliance with national and international data protection and confidentiality regulations (e.g., EU GDPR 2016/679 Recital 26; Swiss HRA, Art. 2 para. 2; Spanish Law 14/2007 on Biomedical Research), all data were fully anonymized before analysis.

### 2.2. Experimental Design

External load was monitored using 106 GPS observations collected across 13 official matches. Data were obtained from players acting as starters (70 observations from 7 matches) and substitutes (36 observations from 9 matches). To ensure data integrity, the following exclusion criteria were applied: (i) only outfield players were monitored, with goalkeepers excluded; (ii) substitutes entering the match during the first half were not considered in the analysis; (iii) players with less than 15 min of playing time were excluded from the analysis; (iv) only players who completed at least one match in both conditions (starter and substitute) were included; and (v) matches in which the team finished with fewer than 11 players were removed from the dataset.

External load was monitored using 10 Hz GPS devices (Apex Pro, STATSports, Newry, Ireland), connected with 20 satellites, which have demonstrated acceptable validity and reliability for measuring movement demands in team sports [22,23]. The GPS signal monitored during data download remained persistent, without compromising the quality of the data. The following variables were obtained: total distance covered (TDC, meters); distance covered between 19.8 and 25.2 km h^−1^ (DC 19.8–25.2 km h^−1^, meters); sprint, defined as distance covered at speeds > 25.2 km·h^−1^ for at least 1 s, terminating when speed falls below 80% of the sprint threshold; number of sprints (#SPR); number of accelerations > 3 m·s^−2^ (#ACC); number of decelerations < 3 m·s^−2^ (#DEC); maximal speed (Vmax, km·h^−1^). Except for Vmax, all variables were expressed as both absolute values and relative values (per minute of exposure). These variables have been widely used in previous research [24,25]. GPS units allocated to starting players were activated by the staff before the players entered the field for the pre-match warm-up, while those assigned to substitutes were activated when their sideline warm-up began. Following match completion, all GPS units were deactivated, and the recorded data were subsequently processed and analyzed by the staff. To minimize inter-unit variability, each player wore the same GPS device throughout the study period. For the purposes of the analysis, players were classified as either starters or substitutes according to their match status.

The team commenced a ~25 min on-field warm-up approximately 40 min before kickoff. Prior to this collective warm-up, each player performed an individualized, self-managed preparation routine in the gym. Only starting players completed the full warm-up protocol, whereas substitutes participated in part of the team-based routine before undertaking an additional individualized warm-up tailored to their anticipated match involvement. The content and temporal sequence of the activities performed by starting players and substitutes are presented in Figure S1. Substitutes were included in the initial team-based warm-up to ensure a minimum level of physical preparedness in the event of an early match entry resulting from injury or other unforeseen circumstances affecting a starting player, while also providing potential psychological benefits associated with inclusion in the team’s pre-match routine [12]. Upon completion of the warm-up, players returned to the dressing room for final preparations. No reactivation exercises were performed upon re-entering the field prior to kick-off.

In contrast to the standardized pre-match warm-up completed by the starting players, the preparatory activities performed by substitutes varied according to the timing of their anticipated match entry. Nevertheless, the interval between the completion of the final preparatory activity and entering the field never exceeded 4 min. To accommodate the inherent unpredictability of substitution timing, the substitutes’ warm-up followed a cyclical structure (Figure S1). Although both warm-up protocols were designed around the same physiological rationale, practical constraints related to time and space necessitated several modifications to the substitutes’ routine. Throughout both protocols, members of the coaching staff supervised the sessions, providing technical guidance, motivation, and ensuring correct execution of all exercises. In accordance with previously established methodologies [18,26], external load during the first 15 min of match play following the warm-up was monitored in the same players under both conditions (as starters and as substitutes), allowing within-player comparisons between the two match-entry conditions. For starting players, this period extended from kick-off to the 15th min of match play. For substitutes, external load monitoring began upon entering the field, and data were included only when players completed at least 15 min of match play (e.g., for a player introduced in the 72nd min, external load was quantified from min 72 to 87). Conversely, if a player entered in the 88th min and the match concluded in the 95th min, the observation was excluded because the player did not complete the required 15 min of match play, including additional time. The mean match-entry time for substitutes was 65 ± 10 min. Consistent with previous studies, the 15 min analysis period was further divided into three consecutive 5 min intervals (0–5, 5–10, and 10–15 min), and external-load variables were compared between starters and substitutes within each interval [8,12]. Stoppage time, extra time, and temporary interruptions of play were not accounted for in either the 15 min or 5 min block analyses. Finally, external-load values across the three 5 min intervals were examined separately according to match-entry status (starters vs. substitutes).

### 2.3. Statistical Analysis

Data are reported as mean ± standard deviation (SD). Normality of the data was assessed using the Shapiro–Wilk test. Differences between starter and substitute conditions were analysed using paired-samples t-tests. The coefficient of variation (CV) was calculated for each player as (SD/mean × 100) across warm-up observations within each condition and then averaged to characterize within-player variability. Differences in running-performance variables across the three consecutive 5 min intervals within each group were assessed using a one-way repeated-measures ANOVA for normally distributed variables. Significant main effects were further examined using Bonferroni-adjusted pairwise comparisons. Statistical significance was established at *p* < 0.05. All analyses were conducted using SPSS software (version 22.0; SPSS Inc., Chicago, IL, USA). Standardized effect sizes (paired Cohen’s d) were calculated and interpreted using the thresholds proposed by Hopkins [27]: trivial (0.00–0.19), small (0.20–0.59), moderate (0.60–1.19), large (1.20–1.99), and very large (≥2.00).

## 3. Results

### 3.1. Comparison Between Starter vs.Substitute Warm-Up

The comparison of absolute and relative external loads of the warm-up activities performed by starting and substitute players is presented in Table 1 and Figure 1. Significant differences were observed between the warm-up protocols performed by starters and substitutes across multiple external load variables. The substitute warm-up was characterized by a significantly shorter duration, lower TDC in both absolute and relative terms, and a lower Vmax (all *p* < 0.001; *d* range = −6.6 to −1.4). Similarly, substitutes performed significantly fewer #ACC in absolute terms (*p* < 0.001; *d* = −1.6), whereas no significant differences were observed in relative #ACC or in #DEC expressed in either absolute or relative terms (*p* > 0.05; *d* range = −0.7 to 0.28). Conversely, DC 19.8–25.2 km·h^−1^ was significantly greater during the substitutes’ warm-up in both absolute and relative terms (both *p* < 0.05; *d* range = 1.3 to 3.6). No significant differences were observed in sprint distance (sprintD) or the number of sprints (#SPR), expressed in either absolute or relative terms (all *p* > 0.05; *d* range = −0.5 to −0.3).

**Table 1:** Comparison of absolute and relative running demands of warm-up activities performed by starters and substitutes.

Running Performance		Starters Warm-Up	Substitutes Warm-Up	*p* Value	*d* (Lower; Upper)
Duration (min)		22.8 ± 1.58	17.0 ± 5.92	<0.001	−3.5 (−5.4; −1.5)
TDC	Absolute (m)	1771.2 ± 133.65	838.1 ± 240.14	<0.001	−6.6 (−7.6; −5.7)
	Relative (m·min^−1^)	77.8 ± 4.50	53.6 ± 12.78	<0.001	−5.1 (−6.5; −3.7)
#ACC	Absolute (#)	20.6 ± 5.00	12.1 ± 3.49	<0.001	−1.6 (−2.1; −1.2)
	Relative (#·min^−1^)	0.9 ± 0.21	0.8 ± 0.18	0.07	−0.7 (−1.5; 0.1)
#DEC	Absolute (#)	9.5 ± 4.11	7.51 ± 3.28	0.08	−0.5 (−0.1; 0.1)
	Relative (#·min^−1^)	0.4 ± 0.19	0.5 ± 0.20	0.52	0.3 (−0.6; 1.2)
DC 19.8–25.2 km·h^−1^	Absolute (m)	37.2 ± 8.04	48.5 ± 20.63	0.03	1.3 (0.1; 2.6)
	Relative (m·min^−1^)	1.6 ± 0.38	3.1 ± 1.42	<0.001	3.6 (1.6; 5.5)
SprintD	Absolute (m)	1.4 ± 1.62	0.46 ± 1.47	0.15	−0.5 (−1.3; 0.2)
	Relative (m·min^−1^)	0.1 ± 0.07	0.04 ± 0.13	0.55	−0.3 (−1.4; 0.8)
#SPR	Absolute (#)	0.2 ± 0.31	0.1 ± 0.25	0.20	−0.4 (−1.1; 0.3)
	Relative (#·min^−1^)	0.01 ± 0.01	0.01 ± 0.02	0.58	−0.3 (−1.3; 0.7)
V_max_ (km·h^−1^)		25.1 ± 0.95	23.8 ± 1.18	<0.001	−1.4 (−2; −0.7)

All data are expressed as mean ± standard deviation; *d* = Cohen’s effect size; m = meters; # = number; TDC = total distance covered; #ACC = number of accelerations > 3 m·s^−2^; #DEC = number of decelerations < 3 m·s^−2^; DC 19.8–25.2 km·h^−1^ = distance covered between 19.8 and 25.2 km·h^−1^; sprintD = distance covered above 25.2 km·h^−1^; #SPR = number of sprint; Vmax = maximal speed.

**Figure 1:**
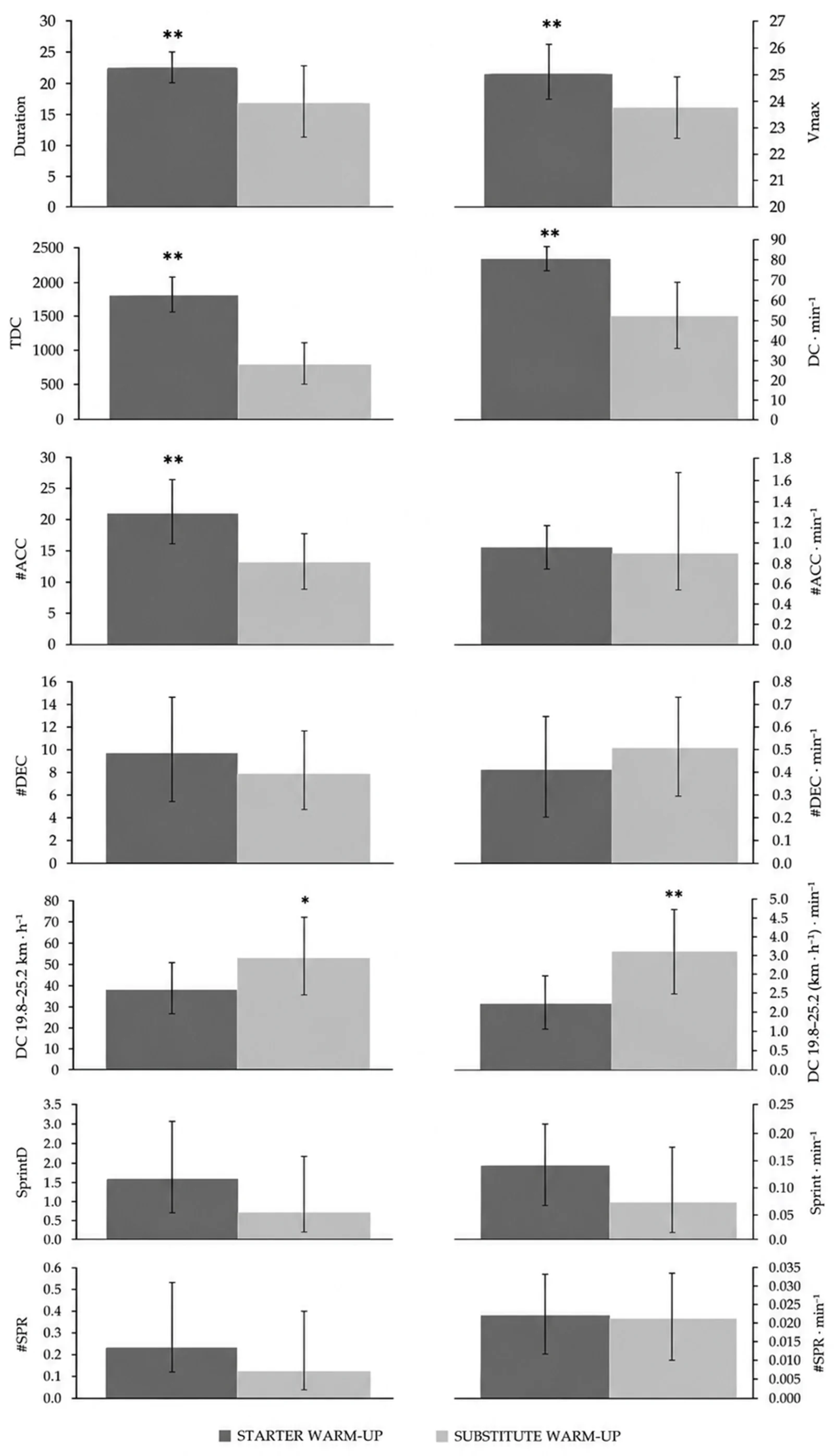
Comparison of absolute and relative running demands of warm-up activities performed by starters and substitutes. TDC = total distance covered; #ACC = number of accelerations > 3 m·s^−2^; #DEC = number of decelerations < 3 m·s^−2^; DC 19.8–25.2 km· h^−1^ = distance covered between 19.8 and 25.2 km·h^−1^; sprintD = distance covered above 25.2 km·h^−1^; #SPR: number of sprint; Vmax = maximal speed; TDC·min^−1^ = total distance covered·min^−1^; #ACC·min^−1^ = number of accelerations > 3 m·s^−2^·min^−1^; #DEC·min^−1^ = number of decelerations < 3 m·s^−2^·min^−1^; DC 19.8–25.2 (km· h^−1^)·min^−1^ = distance covered between 19.8 and 25.2 (km·h^−1^)·min^−1^; sprintD ·min^−1^ = distance covered above 25.2 (km·h^−1^)·min^−1^. * *p* < 0.05; ** *p* < 0.001.

### 3.2. Post Warm-Up Progression Between Starters and Substitutes Across Three 5-Min Periods

The comparison of external load between starters and substitutes during the first 15 min following the two warm-up protocols, including the overall 15 min period and the three consecutive 5 min intervals, is presented in Table 2 and Figure 2. Overall, no significant differences were observed between starters and substitutes in most running variables during the overall 0–15 min period (all *p* > 0.05; *d* range = −0.3 to 0.75). However, when the 15 min period was divided into three consecutive 5 min intervals, several time-specific differences were observed. Starters covered a significantly greater TDC during the 5–10 min interval (*p* < 0.05; *d* = −0.93), whereas no significant differences were identified during 0–5 or 10–15 min intervals (*p* > 0.05; *d* range = 0.09 to −0.13). Conversely, substitutes performed significantly more #ACC during the 10–15 min interval and #DEC, DC 19.8–25.2 km·h^−1^ and sprintD during the initial 0–5 min interval (*p* < 0.05; *d* range = 0.5 to 1.24) with no between-groups differences detected during the subsequent 5–10 or 10–15 min intervals (*p* > 0.05; *d* range = −0.26 to 0.21). No statistical differences were observed in #SPR (all *p* > 0.05; *d* range = −0.60 to 0.24) or Vmax (all *p* > 0.05; *d* range = −0.07 to 0.61) between starters and substitutes across any of the 5 min intervals.

**Table 2:** Comparative analysis of progression between starters and substitutes across three consecutive 5 min periods following the warm-up.

Running Performance	Period	Starters Mean ± SD (m)	Substitutes Mean ± SD (m)	*p* Value	*d*
TDC	0–15	1804.8 ± 180.31	1748.4 ± 153.66	0.18	−0.30
	0–5	593.91 ± 56.33	603.66 ± 56.33	0.60	0.09
	5–10	641.75 ± 53.64	589.28 ± 84.56	0.02	−0.93
	10–15	569.85 ± 72.77	559.69 ± 73.3	0.71	−0.13
#ACC	0–15	13.53 ± 4.88	15.95 ± 3.14	0.09	0.47
	0–5	4.17 ± 2.25	5.67 ± 1.84	0.12	0.63
	5–10	5.51 ± 2.53	5.64 ± 1.38	0.87	0.05
	10–15	3.86 ± 1.66	4.85 ± 1.91	0.01	0.57
#DEC	0–15	16.4 ± 4.09	16.40 ± 4.61	0.99	0.00
	0–5	4.55 ± 1.93	5.57 ± 1.89	0.02	0.50
	5–10	6.61 ± 0.44	5.99 ± 2.34	0.44	−0.24
	10–15	5.30 ± 1.56	4.87 ± 1.72	0.41	−0.26
DC 19.8–25.2 km·h^−1^	0–15	128.10 ± 35.90	133.38 ± 47.81	0.70	0.14
	0–5	38.51 ± 17.78	53.24 ± 21.05	0.01	0.79
	5–10	45.90 ± 25.78	39.40 ± 23.81	0.53	−0.24
	10–15	43.69 ± 17.96	41.63 ± 16.79	0.71	−0.11
SprintD	0–15	23.24 ± 13.49	33.95 ± 21.68	0.09	0.75
	0–5	5.83 ± 5.72	13.29 ± 10.67	0.01	1.24
	5–10	7.48 ± 6.20	8.25 ± 11.10	0.76	0.12
	10–15	9.89 ± 10.00	12.09 ± 13.16	0.60	0.21
#SPR	0–15	1.95 ± 1.09	2.52 ± 2.16	0.27	0.50
	0–5	0.57 ± 0.57	0.71 ± 0.75	0.51	0.24
	5–10	0.73 ± 0.56	0.38 ± 0.66	0.05	−0.60
	10–15	0.63 ± 0.64	0.76 ± 0.76	0.64	0.18
V_max_	0–15	29.47 ± 2.31	30.31 ± 4.98	0.57	0.35
	0–5	26 ± 2.54	27.62 ± 2.22	0.08	0.61
	5–10	26.29 ± 1.75	26.17 ± 2.59	0.83	−0.07
	10–15	26.18 ± 2.71	26.52 ± 2.41	0.68	0.12

All data are expressed as mean ± standard deviation; d = Cohen’s effect size; m = meters; TDC = total distance covered; #ACC = number of accelerations > 3 m·s^−2^; #DEC = number of decelerations < 3 m·s^−2^; DC 19.8–25.2 km·h^-1^ = distance covered between 19.8 and 25.2 km·h^−1^; sprintD = distance covered above 25.2 km·h^−1^; #SPR = number of sprint; Vmax = maximal speed km·h^−1^.

**Figure 2:**
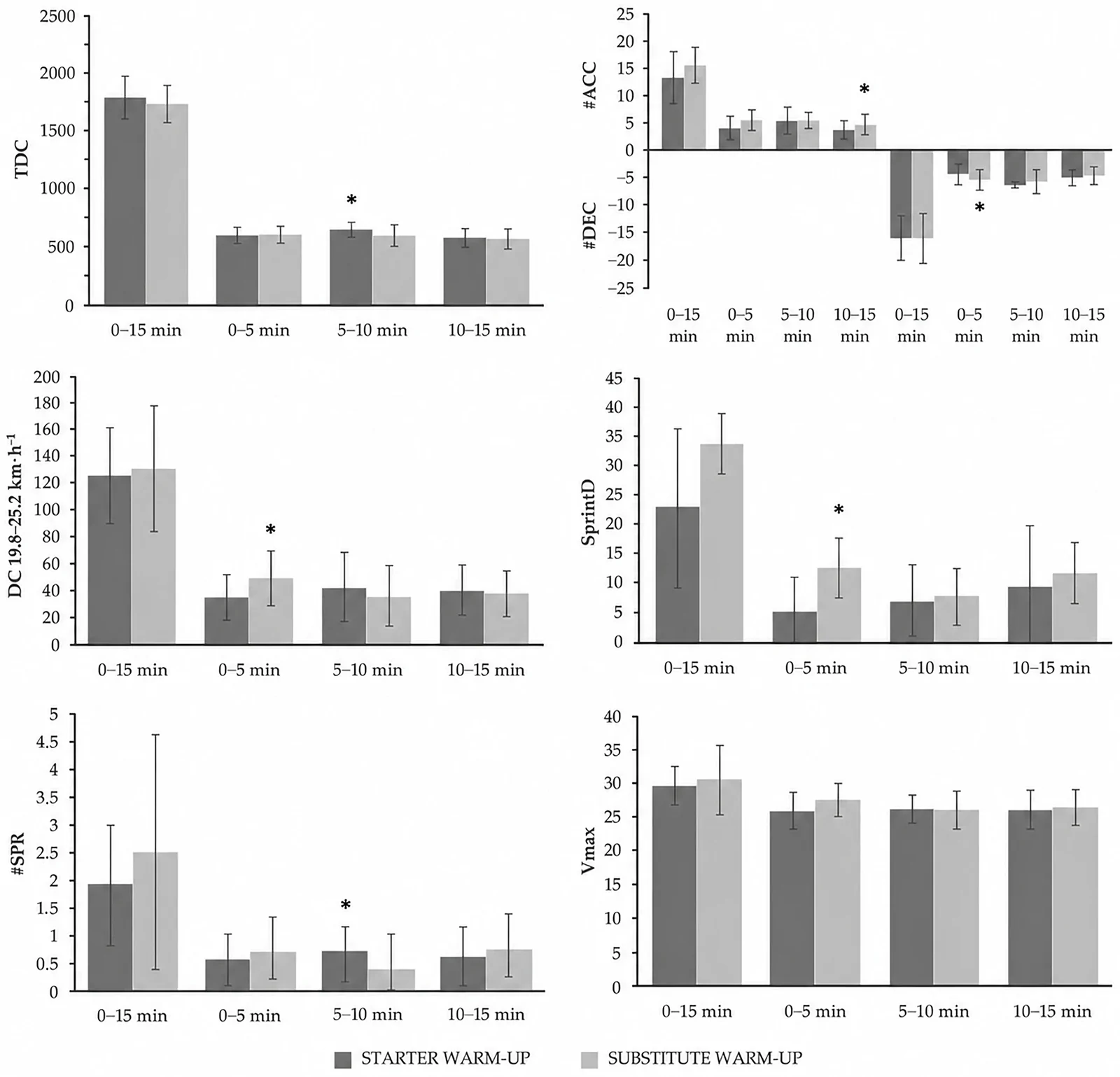
Comparison of the different periods when participating as starters vs. as substitutes. TDC = total distance covered; #ACC = number of accelerations >3 m·s^−2^; #DEC = number of decelerations <3 m·s^−2^; DC 19.8–25.2 km·h^−1^ = distance covered between 19.8 and 25.2 km·h^−1^; sprintD = distance covered above 25.2 km·h^−1^; #SPR: number of sprints; Vmax: maximal speed km·h^−1^. * *p* < 0.05.

### 3.3. Variability of Substitutes and Starters’ Warm-Ups

The variability observed in both warm-up protocols is shown in Table 3. Warm-up duration variability was substantially higher in substitutes than starters. Likewise, TDC exhibited greater variability in substitutes than in starters, both in absolute and relative terms. Regarding accelerations, the variability in absolute #ACC was slightly higher in substitutes than in starters, whereas the variability in relative #ACC was identical between groups. Similarly, absolute #DEC variability was marginally higher in substitutes than in starters, whereas relative #DEC variability was slightly higher in starters than in substitutes. DC19.8–25.2 km·h^−1^ also exhibited greater variability in substitutes than in starters, both in absolute and relative terms. SprintD demonstrated markedly higher variability in substitutes than in starters, both in absolute and relative terms. Consistent with these findings, #SPR also showed substantially greater variability in substitutes than in starters, both in absolute and relative measures. Finally, Vmax exhibited low variability in both groups, although it was slightly higher in substitutes than starters.

## 4. Discussion

The aims of this study were to compare the absolute and relative external load during the warm-up between starting and substitute players, describe the external load accumulated during the first 15 min of match play in both groups following the warm-up, and quantify and compare the between-group variability in warm-up external load. The main finding of this study was that, despite the temporal and spatial constraints associated with preparing substitute players, the substitutes’ warm-up did not differ significantly from that of starters in decelerations and sprint-related variables and included more high-speed running, whereas duration, total distance, accelerations, and maximal speed were lower. Furthermore, although the cumulative external load during the first 15 min of match participation was similar between groups, substitutes performed a greater #DEC, covered more DC at 19.8–25.2 km·h^−1^ and greater sprintD during the first 5 min after entering the match, and performed more #ACC during the 10–15 min interval than starters. Finally, the warm-up performed by substitutes exhibited greater variability across almost all external load metrics than the warm-up performed by the starters.

This study showed that, compared with the warm-up performed by substitutes, the pre-match warm-up performed by starters resulted in greater TDC, expressed in both absolute and relative terms, as well as a higher absolute #ACC. These differences may be primarily explained by the longer duration of the starters’ warm-up, which lasted approximately 6 min longer than the substitutes’ protocol (23 vs. 17.0 min), representing a 34% increase in duration and, consequently, a greater accumulated external load. Although both warm-up protocols included analytical running drills over the same distance (20 m; see Figure S1), the greater #ACC observed in starters may also be explained by the inclusion of more game-specific activities, such as small-sided games, tactical exercises, and position-specific situational drills. These activities require frequent changes in speed and direction and have previously been shown to impose greater acceleration demands than more analytical warm-up tasks [28]. Likewise, starters achieved higher Vmax during the pre-match warm-up than substitutes. Previous studies have shown that the available playing area is a key determinant of players’ ability to reach high running speeds during football-specific activities [29,30]. Indeed, smaller exercise areas consistently result in lower peak velocities than larger training areas or matches [29,31]. Therefore, the restricted space typically available for substitute warm-ups in professional football stadiums may partially explain the lower Vmax attained by these players. Interestingly, despite the shorter duration of their warm-up, substitutes covered greater distance at 19.8–25.2 km·h^−1^ than starters, expressed in both absolute and relative terms. This finding may also be related to the spatial constraints of substitute warm-ups. Specifically, the limited space available may reduce the opportunity to attain higher Vmax or perform tasks that elicit frequent accelerations, leading players to accumulate a greater proportion of their running distance within the high-speed running range rather than at maximal sprinting speeds. These aspects are particularly relevant, as they may directly influence substitutes’ subsequent match performance. Given the unpredictable nature of match play, substitutes may be required to perform a maximal-speed sprint or a high-intensity acceleration immediately after entering the match, despite potentially being inadequately prepared for such demands. Despite these differences, both warm-up protocols elicited similar demands for relative #ACC, absolute and relative #DEC, and sprint variables expressed in both absolute and relative terms. This temporal variation may be related to the distinct contextual and behavioral demands associated with substitute participation. Substitute players are frequently introduced as a tactical intervention to modify match dynamics, increase offensive output, or maintain physical intensity during critical phases of play. Consequently, substitutes may adopt a more aggressive activity profile immediately after entering the match, characterized by greater willingness to perform high-intensity actions and maximize their physical contribution during a limited period of match exposure. Previous research has reported that substitute players typically exhibit greater relative high-intensity running and sprint demands than players who complete the full match, suggesting that playing status may influence the regulation of physical effort during competition [32,33]. Furthermore, the present findings extend previous observations by indicating that this enhanced intensity response is not necessarily maintained throughout the entire initial period of match participation but may instead be expressed within specific short-term phases following match entry. In contrast to previous research examining substitutes introduced later in matches, where accumulated fatigue differences between groups may contribute substantially to performance discrepancies, the present study evaluated only the first 15 min of match participation, suggesting that the observed differences may reflect not only physiological freshness but also the immediate behavioral response associated with entering the match. These findings suggest that, despite the temporal and spatial constraints faced by practitioners when preparing substitute players, the warm-up protocol adopted provides an external load profile broadly comparable to that of the starters’ pre-match warm-up across several key locomotor variables.

The notion that the warm-up performed by substitutes is comparable to that of starters is further supported by the absence of between-group differences in any running performance variable during the overall first 15 min of match play. The comparable external load achieved by both warm-up protocols, together with the similar running demands observed during the first 15 min of match play, may suggest that both protocols may provide an adequate mechanical stimulus before match entry. However, these findings provide information only on players’ physical performance and do not allow conclusions to be drawn regarding the overall impact of the substitute warm-up on match performance. In particular, the absence of ball-related activities during the substitute warm-up may have limited players’ preparation for the technical and perceptual demands of match play, potentially influencing their involvement in the game during the initial stages after entering the match. Our results contrast with previous studies reporting that substitute players cover greater total distance, high-intensity running distance, and sprint distance than both the players they replace and those who complete the entire match [33,34,35]. These discrepancies between studies may be explained by methodological differences. Previous research [33,35] quantified external match load using relative metrics and analyzed players’ entire match participation, whereas the present study specifically examined the cumulative external load during the first 15 min of play to isolate the potential influence of the warm-up. What does appear to be consistent with previous studies is the players’ response when the initial 15 min period is divided into three consecutive 5 min intervals, during which differences between players emerge according to playing status. During the initial 0–5 min interval, substitutes performed more #DEC, covered greater distance at 19.8–25.2 km·h^−1,^ and sprint distance than starters. During the 5–10 min interval, starters covered greater total distance. Moreover, substitutes performed more #ACC during the 10–15 min interval. A previous study by the same research group, which analyzed the responses of substitutes and starters during the initial minutes of match play using a similar sample, reported contrasting findings. Specifically, in the previous study, starters covered greater total distance during the first 15 min, as well as during the 5–10 and 10–15 min intervals compared with substitutes, whereas no differences between groups were observed in high-speed running or sprint distance [15]. The discrepancies between studies, together with the different external load patterns observed between substitutes and starters when match external load demands were analyzed in consecutive 5 min intervals, could be explained by the tactical and psychological contextual factors surrounding player substitutions. Coaches often introduce substitutes to alter match dynamics or provide a fresh physical stimulus, which may encourage them to adopt a more aggressive pacing immediately after entering the match, in an attempt to rapidly influence the game or justify their selection [36]. Although these interpretations remain largely based on empirical observation rather than robust scientific evidence, further research is needed to better understand the role and performance of substitute players in football [37].

Another objective of the present study was to examine the variability associated with the two warm-up modalities. The findings demonstrated that warm-up variability depends on both player status and the external load variable considered. Specifically, the CV for #ACC, #DEC, and Vmax were comparable between starting and substitute players (~25%, ~45%, and ~4–5%, respectively). In contrast, total distance, high-speed running distance, sprint distance, and number of sprints exhibited substantially greater variability in substitutes (>20%, >40%, >300%, and ~400%, respectively) than in starters (~6–7%, >20%, >115%, and ~150%, respectively). This greater variability may be partially explained by the longer and more heterogeneous warm-up duration performed by substitutes, as discussed previously. Not only were substitute warm-ups shorter, but their duration also exhibited considerably greater variability, with values up to sevenfold higher than those observed in starters. In addition, substitutes have been shown to perform up to three re-warm-up bouts during a single match [12], which may further contribute to the greater variability observed in this group. Another important explanation is the inherently less predictable nature of substitute preparation, which is likely influenced by contextual factors such as match dynamics, substitution timing, tactical requirements, and coaching decisions [12,36]. In contrast, starters generally complete a structured and standardized pre-match warm-up, resulting in lower between-player variability [36]. Furthermore, variability increased with movement intensity, with sprint-related variables displaying the greatest fluctuations among substitutes, whereas maximal running speed remained relatively consistent in both groups. This finding suggests that, although players may achieve similar peak running speed during warm-up, the volume and frequency of high-intensity actions performed before match entry are considerably more variable, particularly among substitutes [38]. For sprint-related variables, CV values exceeding 300% indicate that the standard deviation was more than three times the mean, reflecting the high variability associated with the low frequency of sprinting efforts and the large proportion of values close to zero. Consequently, the absence of statistically significant differences in sprint distance and #SPR between starters and substitutes (all *p* > 0.05) should be interpreted as an absence of detectable differences rather than evidence of equivalence. Furthermore, the corresponding effect sizes should be interpreted with caution due to the high variability observed in these measures. From a practical perspective, sprint exposure during substitutes’ warm-ups cannot be assumed to be consistent or representative across players. Therefore, individualized monitoring may be more appropriate for assessing whether each substitute has received sufficient high-intensity running exposure before entering the match. These differences may have important implications for match readiness, as the physiological and neuromuscular stimuli induced before match entry are determined not only by the attainment of maximal running intensity but also by the accumulated volume of high-intensity activities performed during the warm-up [38]. Previous research on warm-up variability has predominantly focused on highly standardized protocols and primarily examined internal-load responses, with limited evidence regarding the variability of external-load demands during elite football warm-ups, particularly among substitute players [34]. For example, a professional football warm-up protocol reported CV values ranging from 1.4% to 7.0% for heart-rate responses [39], whereas studies investigating the FIFA 11+ programme have reported similarly low day-to-day variability in heart rate (CV = 0.45–2.57%) and ratings of perceived exertion (CV = 5.07%) [40]. However, direct comparisons with the present findings should be made with caution. These standardized protocols are characterized by fixed exercise sequences, predefined durations, controlled exercise volumes, and the absence of match-specific activities. Conversely, the competitive warm-ups analyzed in the present study were designed to incorporate sport-specific movements and activities that replicate match-related demands, particularly for starters, thereby providing a more ecologically valid context than laboratory-based or highly standardized protocols.

Although the present study provides novel information on the external load demands of professional soccer players following starter and substitute warm-up routines, several limitations should be considered. First, the relatively small sample and individual player fitness levels, drawn from a single professional team and restricted to external-load measures, may limit the transferability of the findings. Future studies should include larger samples from multiple teams and competitive levels while also incorporating internal-load and physiological measures to better understand the mechanisms underlying the differences in pre-match preparation between starters and substitutes. Second, playing position and tactical roles were not considered, precluding position-specific analyses. Investigating whether warm-up external-load profiles differ according to playing position would provide valuable insights into whether pre-match and pre-entry preparation should be tailored to the specific physical and tactical demands of each playing position. Finally, contextual factors such as match status, opposition quality, tactical role, and substitution timing were not considered, despite their potential influence on warm-up load and preparation strategies. In addition, GPS-derived variables quantify external load but do not directly evaluate players’ performance, as they do not capture technical, tactical, or effectiveness-related aspects of match play. Therefore, it cannot be assumed that both warm-up protocols had the same impact on players’ performance during match play. Accordingly, future studies should account for these factors to better understand how contextual constraints shape warm-up practices and to optimize pre-entry preparation strategies for both starters and substitutes.

## 5. Conclusions

The present study demonstrated that starting and substitute players exhibited distinct pre-match warm-up external-load profiles, with starters accumulating greater warm-up duration, total distance, acceleration demands, and exposure to maximal running speed. Nevertheless, substitutes achieved greater exposure to high-speed running and comparable values for several other high-intensity locomotor variables, indicating that their shorter and more constrained warm-up may provide an adequate mechanical stimulus before match entry. During the first 15 min of match participation, no between-groups differences were observed in cumulative external load. However, substitutes performed greater high-intensity actions during specific 5 min intervals following match entry. Furthermore, substitute warm-ups exhibited greater variability, particularly for sprint-related variables, reflecting the contextual constraints associated with substitute preparation compared with the more consistent pre-match routine performed by starters. These findings suggest that current substitute warm-up strategies may provide adequate preparation for the early demands of match play. However, greater consistency in external-load exposure may further optimize pre-entry preparation in elite football.

The findings of the present study have several practical implications for the design and monitoring of pre-match and pre-entry warm-up strategies in professional soccer. Despite the temporal constraints experienced by substitutes, their warm-up protocols generated locomotor demands comparable to those of starters for several key variables. However, starters accumulated greater total external load, total distance, and acceleration demands owing to their longer and more match-specific preparation. Therefore, substitute warm-ups should prioritize high-intensity activities, including accelerations, decelerations, and sprint exposure, to reproduce key physical stimuli within the limited time available. Considering the present findings, providing substitutes with a larger warm-up area during matches, in terms of both length and width, may allow them to better replicate the physical demands of starters’ warm-ups. In particular, a larger area could facilitate greater exposure to high-speed running and potentially maximal sprinting velocities, while also allowing for more frequent accelerations through football-specific activities, such as small-sided games. As an alternative, when such space is not available, the half-time interval could be used to implement ball possession games or small-sided games for substitutes, providing football-specific physical stimuli that might otherwise be lacking during the prolonged period before match entry.

The comparable external load observed during the initial 15 min of match participation suggests that current substitute warm-up strategies may provide adequate preparation for the early phases of match play. Nevertheless, the greater frequency of high-intensity actions performed by substitutes after entering the match indicates that a final re-warm-up immediately before entry may be beneficial, particularly when players are expected to fulfil physically demanding tactical roles. Incorporating explosive, match-specific activities immediately before substitution may therefore enhance readiness for the high-intensity demands typically experienced upon entering the game.

Finally, the substantially greater variability observed in substitute warm-ups highlights the importance of systematic monitoring and, where feasible, greater standardization of pre-entry preparation. Although complete standardization is unlikely to be achievable given the contextual constraints surrounding player substitutions, real-time external-load monitoring may help practitioners ensure that substitutes receive an appropriate and consistent mechanical stimulus before entering the match. Such an approach may improve substitute readiness while increasing the consistency and effectiveness of pre-entry warm-up practices in elite soccer.

## Figures and Tables

**Table 3:** Variation in absolute and relative running demands of warm-up activities performed by starters and substitutes.

Running Performance		CV Starter Warm-Up *(%)*	CV Substitute Warm-Up *(%)*
Duration (min)		6.9	34.8
TDC	Absolute (m)	7.5	28.7
	Relative (m·min^−1^)	5.8	23.8
#ACC	Absolute (n°)	24.2	28.9
	Relative (m·min^−1^)	23.5	23.5
#DEC	Absolute (n°)	43.2	43.7
	Relative (n°·min^−1^)	44.3	42.6
DC 19.8–25.2 km·h^−1^	Absolute (m)	21.6	42.5
	Relative (m·min^−1^)	23.4	46.1
SprintD	Absolute (m)	118.3	317.9
	Relative (m·min^−1^)	116.7	348.4
#SPR	Absolute (n°)	150.4	399.9
	Relative (n°·min^−1^)	153.1	399.3
V_max_ (km·h^−1^)		3.8	5

All data are expressed as %; CV = Coefficient of Variation; m = meters; n° = number; TDC = total distance covered; #ACC = number of accelerations > 3 m·s^−2^; #DEC = number of decelerations < 3 m·s^−2^; DC 19.8–25.2 km·h^−1^ = distance covered between 19.8 and 25.2 km·h^−1^; sprintD = distance covered above 25.2 km·h^−1^; #SPR = number of sprint; Vmax = maximal speed.

## Data Availability

The raw data supporting the conclusions of this article will be made available by the authors upon request.

## Supplementary Materials

The following supporting information can be downloaded at: https://www.mdpi.com/article/10.3390/jfmk11040432/s1. Figure S1: Starters and substitutes warm-up before kick-off and after half-time. Min = exercise duration in minutes.
