# Supplementary material for: External Load During Warm-Up and Early-Match Running Performance of Starting and Substitute Football Players: A Within-Player Comparison
Source: J Funct Morphol Kinesiol. 2026 Oct 19;11(4):432. doi: 10.3390/jfmk11040432 (PMC13692504; doi:10.3390/jfmk11040432)
Supplement: Supplementary file 1 [file jfmk-11-00432-s001.zip › jfmk-4578862-supplementary.pdf]

SUBSTITUTES	STARTERS	
free movement: players moved freely on and off the ball	free movement: players moved freely on and off the ball	2 min.
dynamic mobility and running drills over a 10-meter distance, concluding with two 10-meter shuttle runs and two 40 –meter accelerations	dynamic mobility and running drills over a 10-meter distance, concluding with two 10-meter shuttle runs and two 40 –meter accelerations	4 min.
technical drills	passing drills on a 9x10 meter square	4 min.
	possession play 5v5 on an 18x21 meter surface, with three neutral support players to encourage ball	5 min.
	tactical work, players performed position-specific tactical movements reflecting match scenarios	5 min.
assisted the starters	specific situational exercises, attacking phase (ball-related and defensive phase	3 min.
	three 20–meter sprints preceded by 5 single–leg and 5 double–leg plyometric jumps forward	2 min.

players returned to the dressing room for final preparations. No reactivation exercises were performed upon re-entering the field prior to kick -off

Kick-off

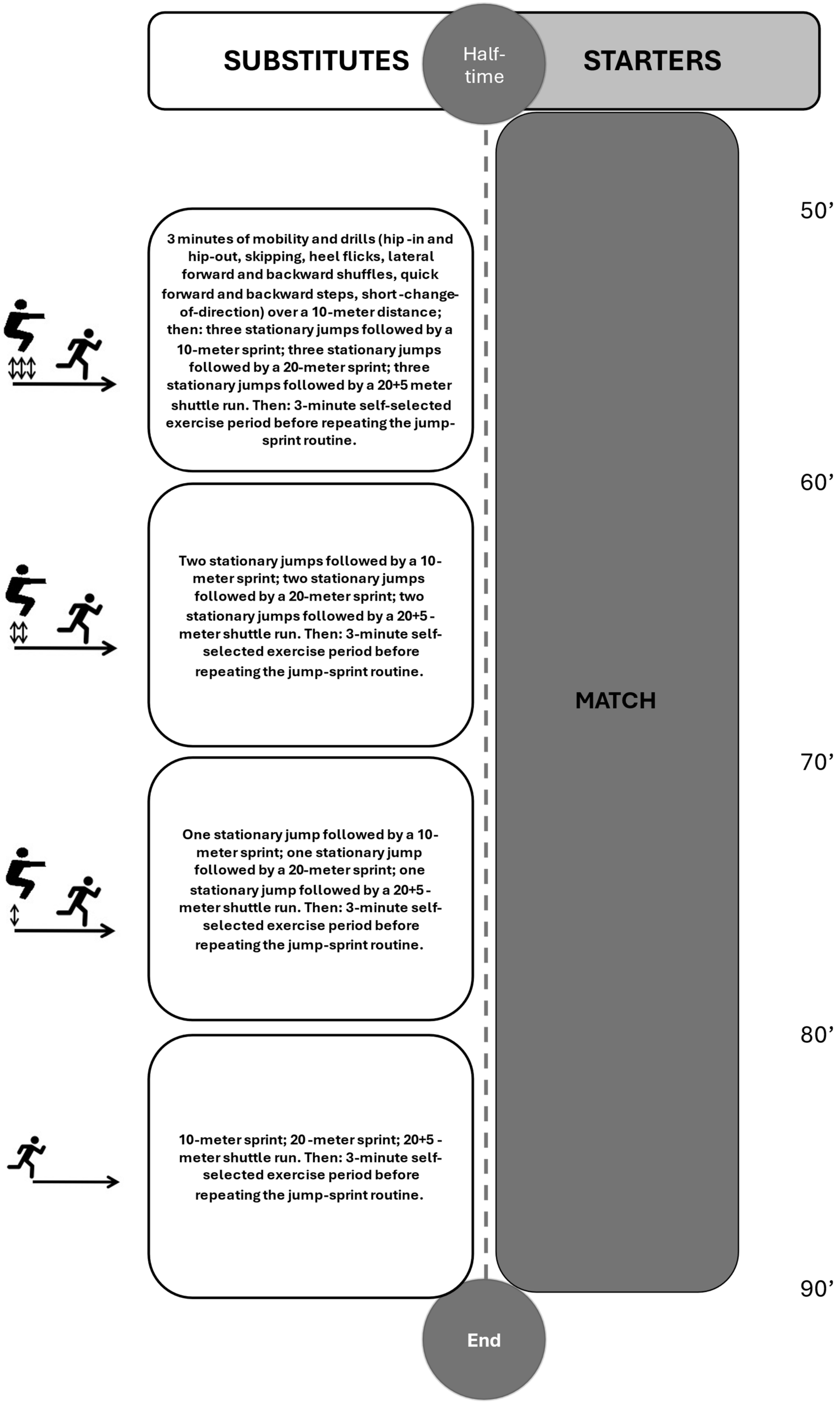
SUBSTITUTES
Half-time
STARTERS
50’
3 minutes of mobility and drills (hip -in and hip-out, skipping, heel flicks, lateral forward and backward shuffles, quick forward and backward steps, short -change-of-direction) over a 10-meter distance; then: three stationary jumps followed by a 10-meter sprint; three stationary jumps followed by a 20-meter sprint; three stationary jumps followed by a 20+5 meter shuttle run. Then: 3-minute self-selected exercise period before repeating the jump-sprint routine.
60’
Two stationary jumps followed by a 10-meter sprint; two stationary jumps followed by a 20-meter sprint; two stationary jumps followed by a 20+5 -meter shuttle run. Then: 3-minute self-selected exercise period before repeating the jump-sprint routine.
MATCH
70’
One stationary jump followed by a 10-meter sprint; one stationary jump followed by a 20-meter sprint; one stationary jump followed by a 20+5 -meter shuttle run. Then: 3-minute self-selected exercise period before repeating the jump-sprint routine.
80’
10-meter sprint; 20 -meter sprint; 20+5 -meter shuttle run. Then: 3-minute self-selected exercise period before repeating the jump-sprint routine.
90’
End

Figure S1. *Starters and substitutes warm-up before kick-off and after half time. Min = exercise duration in minutes.*
